# Melatonin increases chilling tolerance in postharvest peach fruit by alleviating oxidative damage

**DOI:** 10.1038/s41598-018-19363-5

**Published:** 2018-01-16

**Authors:** Shifeng Cao, Jiarong Shao, Liyu Shi, Liwei Xu, Ziming Shen, Wei Chen, Zhenfeng Yang

**Affiliations:** College of Biological and Environmental Sciences, Zhejiang Wanli University, Ningbo, 315100 People’s Republic of China

## Abstract

Melatonin has been reported to alleviate chilling symptoms in postharvest peach fruit during cold storage, however, the mechanism involved is largely unknown. To better understand its role in chilling tolerance, here we investigated the effects of melatonin on oxidative damage in peach fruit subjected to chilling after harvest. Chilling injury of peaches was dramatically reduced by melatonin treatment. Melatonin induced hydrogen peroxide (H_2_O_2_) content at the early stage of storage but inhibited its accumulation thereafter. Meanwhile, melatonin also up-regulated the expression of genes involved in antioxidant responses in peaches. In addition, compared to the control fruit, peaches treated with melatonin displayed higher transcript abundance of ascorbic acid (AsA) biosynthetic genes and consequently increased the AsA content. Our results suggested that in response to melatonin during chilling, the high H_2_O_2_ level in the treated peaches at the initial time of storage, may work as a signaling molecule to induce protective mechanisms via up-regulating the expression of antioxidative genes and increasing AsA content. On the other hand, after the transient increase in the treated peaches, H_2_O_2_ was efficiently removed because of the activated antioxidant systems, which was associated with the higher chilling tolerance induced by melatonin.

## Introduction

Temperature is one of the most predominant factors for maintaining quality of peach fruit after harvest. Some of metabolic activities such as ripening and decay are reduced by lowering temperatures^1,2^. However, storage at temperature range of 2.2–7.6 °C causes certain physiological disorders known as chilling injury. Classic symptoms of chilling injury in peach fruit include flesh browning, reddening/bleeding and woolliness/mealiness^1,2^. Due to this phenomenon, fruit quality is deteriorated significantly during postharvest storage and the storage life is limited.

The appearance of chilling damage is often associated with oxidative stress resulted from an elevated production of reactive oxygen species (ROS) such as superoxide anion (O_2_^−^), hydrogen peroxide (H_2_O_2_), hydroxyl radical, nitric oxide, and peroxynitrite^3^. Oxidative damage is considered to be an early response of sensitive tissues to chilling^4^. If the production of ROS increases dramatically, as occurs under environmental stress, hydroxyl radical reacts with membrane lipids, which leads to lipid peroxidation and membrane degradation^5^. Malondialdehyde (MDA) is a product of this lipid peroxidation, and is used as an indicator of stress in some tissues^6^. In order to cope with ROS, plants have evolved an efficient antioxidant defense system to respond to oxidative stress and prevent the accumulation of ROS and repair oxidative damage^7^. This system involves both lipid-soluble antioxidants (α-tocopherol and carotene), water-soluble reductants including ascorbate acid (AsA) and glutathione (GSH), and enzymes such as catalase (CAT), ascorbate peroxidase (APX), superoxide dismutase (SOD) and glutathione reductase (GR)^8^.

Melatonin, (N-acetyl-5-methoxytiyptamine), a neural hormone secreted by the pineal gland in mammals, has been found in several plant tissues^9^. It has been reported to be involved in growth, development and stress responses in plants^10–22^. Recently, melatonin has been shown promising in regulating fruit ripening and preventing postharvest disease and disorders in horticultural crops. For example, grape berries treated with melatonin at pre-veraison exhibited higher endogenous melatonin accumulation, which not only increased berry size and weight, but also increased synchronicity of berry ripening^23^. A postharvest application of melatonin effectively delayed senescence and maintained quality of peach fruit stored at ambient temperature^24^. Exogenous melatonin pre-treatment improved anthocyanin accumulation by regulating gene expressions and resulted in high reactive oxygen species scavenging capacity in cabbage sprouts^25^. Treatment with melatonin delayed cassava roots postharvest physiological deterioration, which was resulted from lowering H_2_O_2_ accumulation^26^. The beneficial impacts of melatonin treatment on attenuating postharvest decay has also been demonstrated in strawberry fruit^27^. Additional, a label-free differential proteomics analysis revealed the effect of melatonin on promoting fruit ripening and anthocyanin accumulation upon postharvest in tomatoes^28^.

Our previous work has shown that melatonin could inhibit chilling injury and enhance the defense response against chilling in cold stored peach fruit^29^. However, the underlying physiological and molecular mechanism of melatonin in the induction of tolerance to cold stresses remains unclear. As a positive regulator of anti-ROS process, solid evidence suggests that melatonin can not only directly scavenge some ROS, but can also modulate antioxidant enzymes and enhance cellular antioxidants^30,31^. This study was therefore conducted to investigate whether the mitigating effect of melatonin is associated with its effect on the antioxidant response of postharvest peaches exposed to cold stress.

## Results

### Melatonin increased chilling tolerance in postharvest peaches

**Figure 1:**
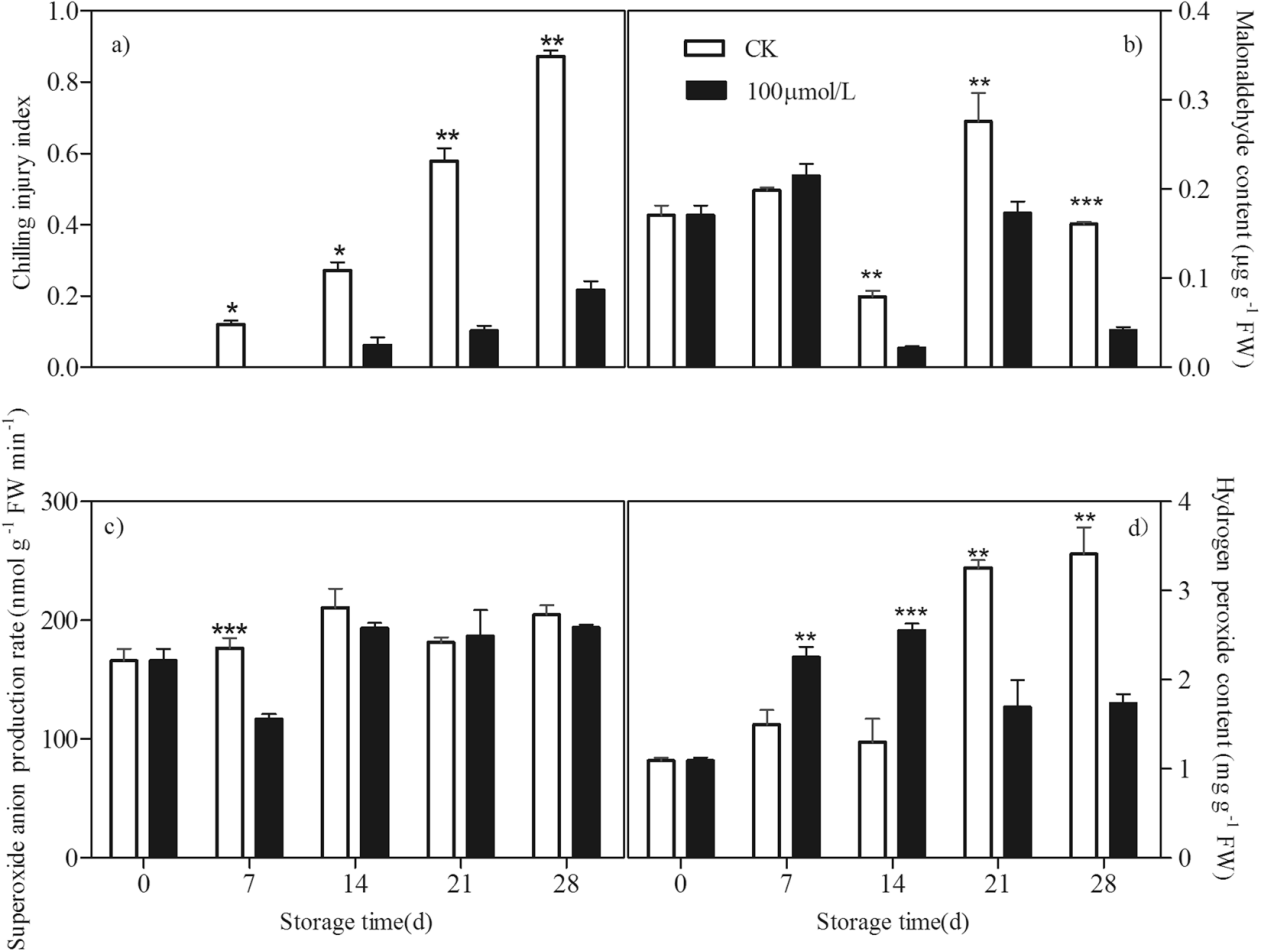
Effect of exogenous melatonin on chilling injury index (**a**) and contents of MDA (**b**), O_2_^−^ (**c**) and H_2_O_2_ (**d**) of peach fruit during storage at 4 °C. All data about the chilling injury index is presented as a mean of fifteen biological replicates and error bars represent ± standard errors. Data about contents of MDA, O_2_^−^ and H_2_O_2_ is presented as a mean of three biological replicates and error bars represent ± standard errors. Asterisks (*) indicate significant differences among melatonin-treated and control samples (Student’s unpaired T test; *P < 0.05, **P < 0.01, and ***P < 0.001).

Compared with the control peaches, melatonin treatment delayed and reduced the chilling injury in peach fruit during the cold storage. Chilling was expressed as internal browning (Fig. 1a). The accumulation of MDA is the marker of lipid peroxidation and damage of cellular membranes. The MDA content of the control fruit decreased during the first 14 days and then sharply increased to a peak at the 21st day before declining. Melatonin treatment significantly inhibited MDA content after 14 days of storage (Fig. 1b).

O_2_^−^ and H_2_O_2_ are major ROS induced by chilling stress. In control fruit, no significant changes in O_2_^−^ content was observed. However, under chilling stress, H_2_O_2_ level accumulated within storage time and a dramatically increase happened at 21st day. As compared to the control fruit, lower O_2_^−^ was found at the 7th day after melatonin treatment (Fig. 1c). However, the treatment induced the increase in H_2_O_2_ content during the first 14 days of storage but inhibited its accumulation thereafter (Fig. 1d).

### Melatonin increased expression of genes involved in antioxidative pathway

**Figure 2:**
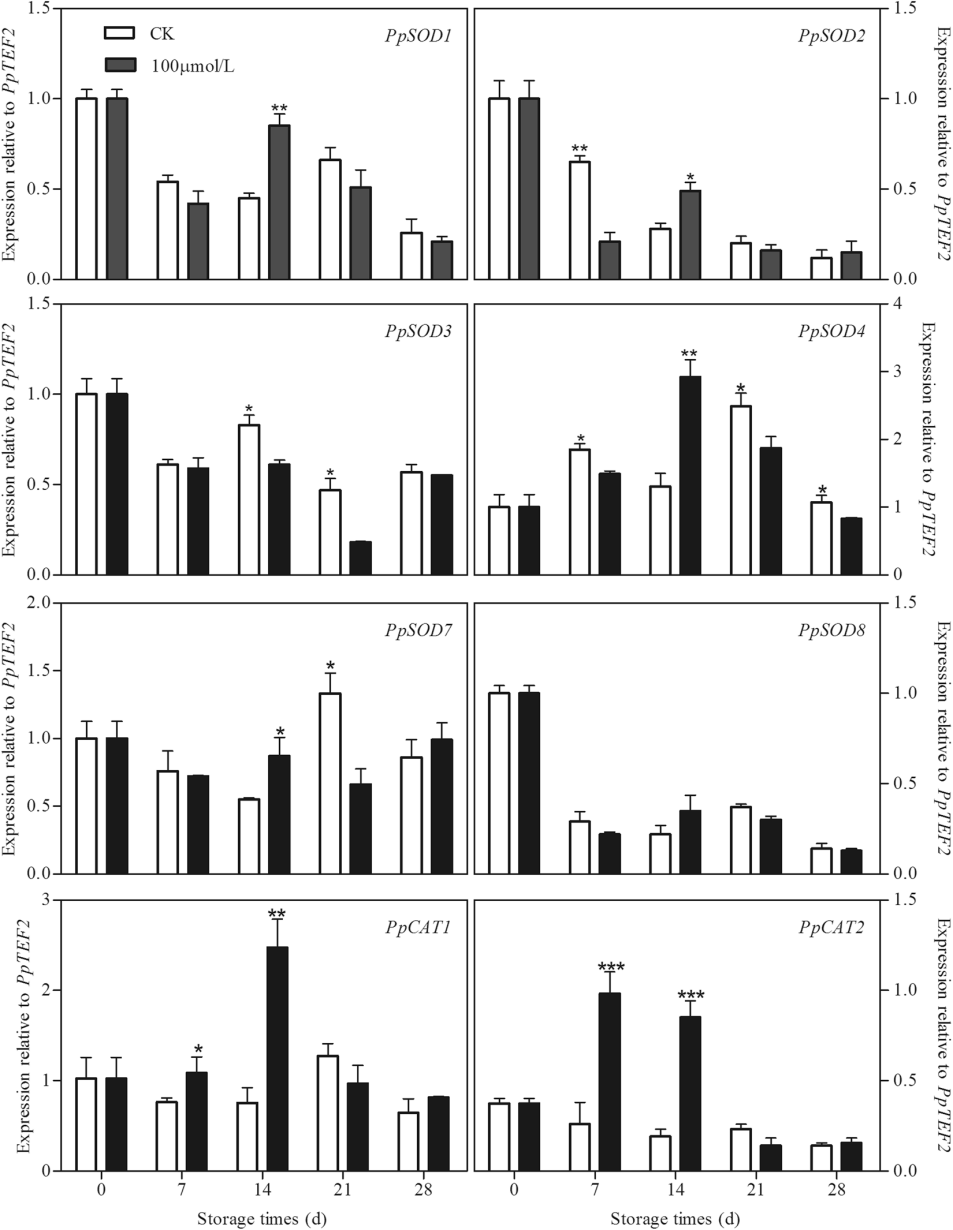
Effect of exogenous melatonin treatment on expression of *PpSOD*s *and PpCAT*s in peach fruit during storage at 4 °C. All data is presented as a mean of three biological replicates and error bars represent ± standard errors. Transcript abundance was determined using qPCR and was normalized using *PpTEF2*. SOD, superoxide dismutase. CAT, catalase. Asterisks (*) indicate significant differences between melatonin-treated and control samples (Student’s unpaired T test; *P < 0.05, **P < 0.01, and ***P < 0.001).

The effect of melatonin application on the transcripts of *PpSOD*s in peach fruit is shown in Fig. 2. Among the 6 *PpSOD*s, the expression of *PpSOD1* and *PpSOD7* in the control peaches decreased during the first 14 days of storage and then increased and reached a peak before a decline. The transcripts of *PpSOD2*, *PpSOD3* and *PpSOD8* decreased along with the storage time, while the expression of *PpSOD4* increased during storage then declined at the end of storage. Melatonin up-regulated *PpSOD1*, *PpSOD2*, *PpSOD4* and *PpSOD7* at the 14th day but inhibited the expression of *PpSOD2* at the 7th day and *PpSOD4* during the whole storage except day 14. In addition, higher expression of *PpSOD3* could also be observed in the control peaches at 14 and 21 days than in treated fruit. There was no significantly difference in *PpSOD8* expression between the control and treated peaches. No significant changes in expression of *PpCAT*s was observed in control peach subjected to chilling during storage. However, in melatonin treated fruit, the expression of *PpCAT1* and *PpCAT2* increased dramatically and reached their peaks at 14th and 7th day of storage, respectively. As compared to the control fruit, higher transcripts of these two *PpCAT*s could be found at 7th and 14th days (Fig. 2).

### Melatonin increased AsA content and upregulated genes involved in ASA-GSH cycle

**Figure 3:**
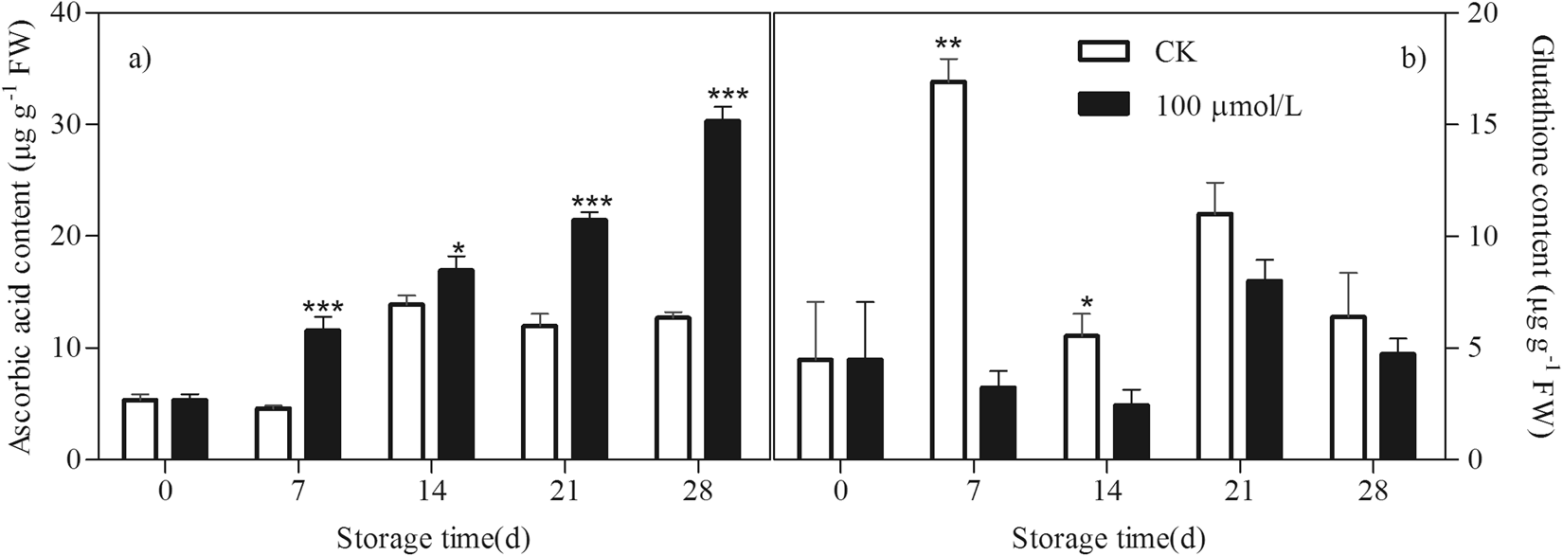
Effect of exogenous melatonin treatment on contents of AsA (**a**) and GSH (**b**) in peach fruit during storage at 4 °C. All data is presented as a mean of three biological replicates and error bars represent ± standard errors. Asterisks (*) indicate significant differences between melatonin-treated and control samples (Student’s unpaired T test; *P < 0.05, **P < 0.01, and ***P < 0.001).

AsA and GSH can directly detoxify ROS and thus contribute to non-enzymatic ROS scavenging. During storage, in the control peaches, the AsA level increased slightly under chilling stress condition. GSH content increased sharply at the first 7 days of storage but declined thereafter. Melatonin treatment induced the AsA accumulation during the whole storage but maintained a lower level of GSH content at 7th and 14th days of storage as compared to the control fruit (Fig. 3).

**Figure 4:**
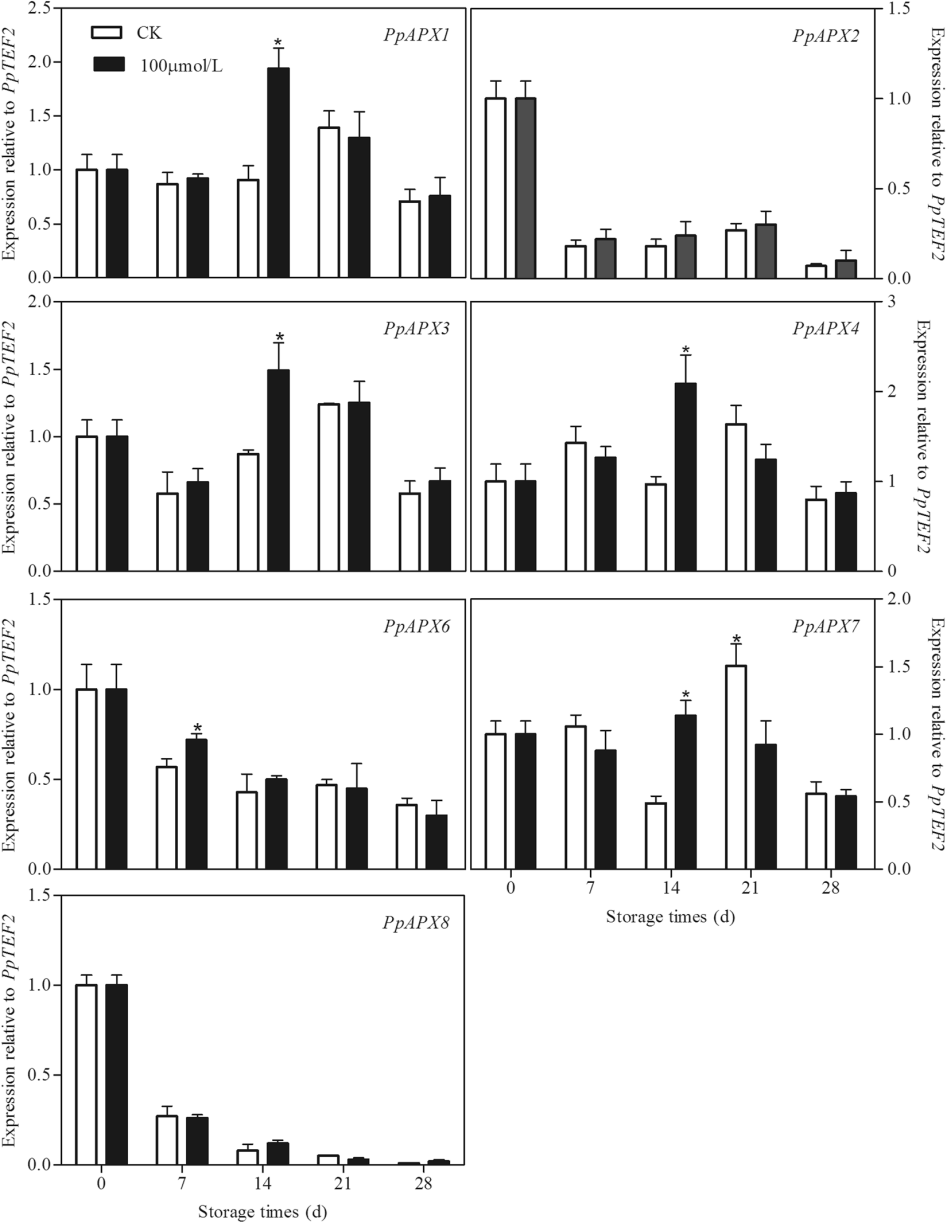
Effect of exogenous melatonin treatment on expression of *PpAPX* genes in peach fruit during storage at 4 °C. All data is presented as a mean of three biological replicates and error bars represent ± standard errors. Transcript abundance was determined using qPCR and was normalized using *PpTEF2*. *APX*, ascorbate peroxidase. Asterisks (*) indicate significant differences between melatonin-treated and control samples (Student’s unpaired T test; *P < 0.05, **P < 0.01, and ***P < 0.001).

**Figure 5:**
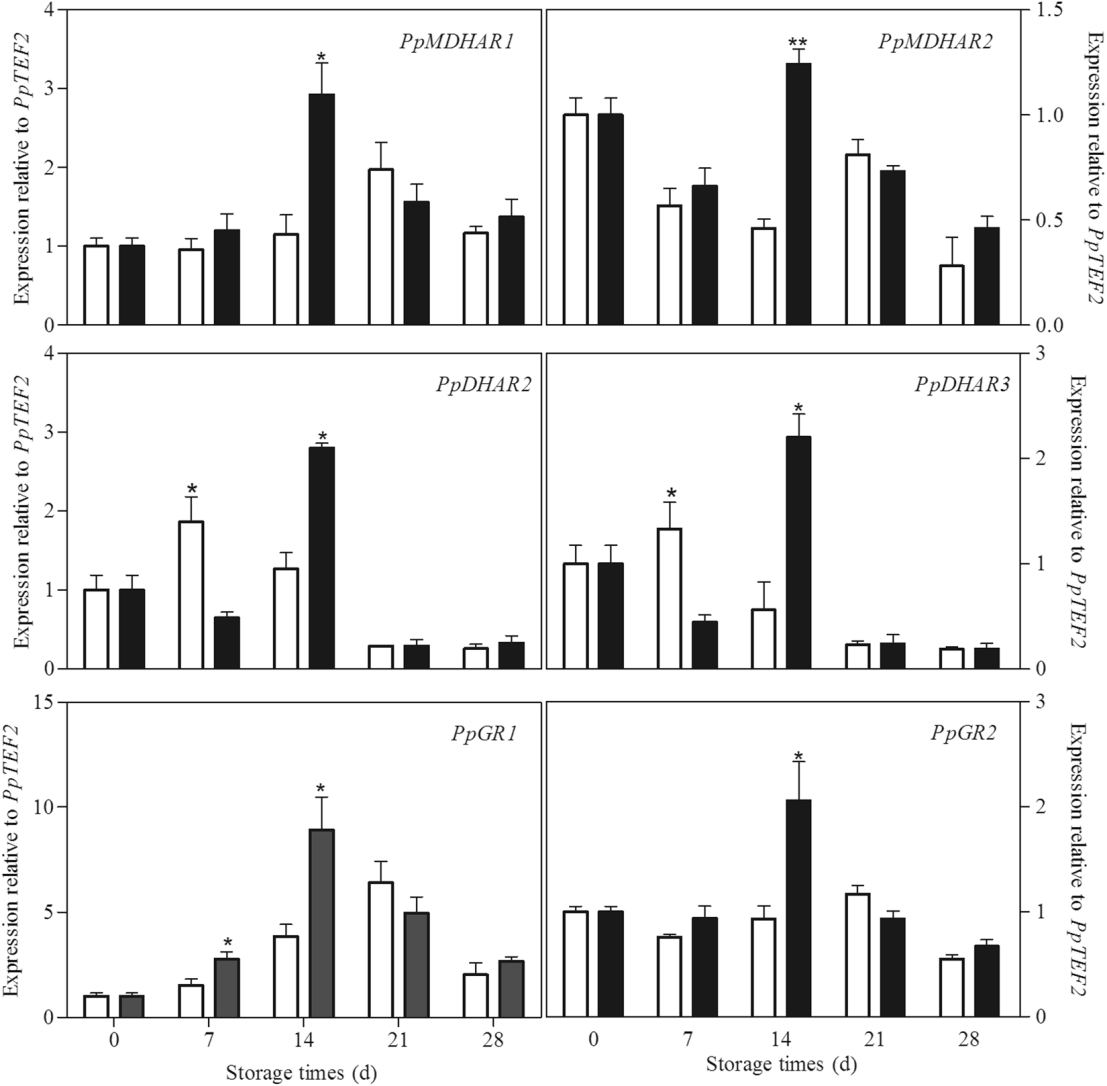
Effect of exogenous melatonin treatment on expression of *PpMDHAR, PpDHAR* and *PpGR* genes in peach fruit during storage at 4 °C. All data is presented as a mean of three biological replicates and error bars represent ± standard errors. Transcript abundance was determined using qPCR and was normalized using *PpTEF2*. *MDHAR*, monodehydroascorbate reductase. *DHAR*, dehydroascorbate reductase. *GR*, glutathione reductase. Asterisks (*) indicate significant differences between melatonin-treated and control samples (Student’s unpaired T test; *P < 0.05, **P < 0.01, and ***P < 0.001).

Seven *PpAPX*s in peach fruit were analysed. As shown in Fig. 4, in the control peaches under chilling stress, *PpAPX2*, *PpAPX6* and *PpAPX8* decreased gradually with the storage, but the other four *APX*s increased firstly and declined at the end of storage. Melatonin treatment induced the expression of *PpAPX1*, *PpAPX3*, *PpAPX4* and *PpAPX7* at 14th days of storage. A higher transcript of *PpAPX6* was found at 7th day in treated peaches as compared to control fruit. There was no significant difference on the *PpAPX2* and *PpAPX8* between the control and treated fruit. The expression of two *PpMDHAR*s in control peaches increased gradually during the first 21 days of storage and then declined. The two *PpDHAR*s also experienced a similar change pattern during storage. The transcripts of the two genes increased at 7th day of storage and then decreased during the remaining storage time. The expression of *PpGR1* increased firstly and then declined at the end of storage. No significant change was observed in the *PpGR2* expression in the control fruit during storage. Interestingly, melatonin treatment up-regulated the transcripts of all the six genes at 14th day, but down-regulated the expression of two *PpDHAR*s at 7th day of storage (Fig. 5).

### Melatonin increased transcript abundance of AsA biosynthetic genes

**Figure 6:**
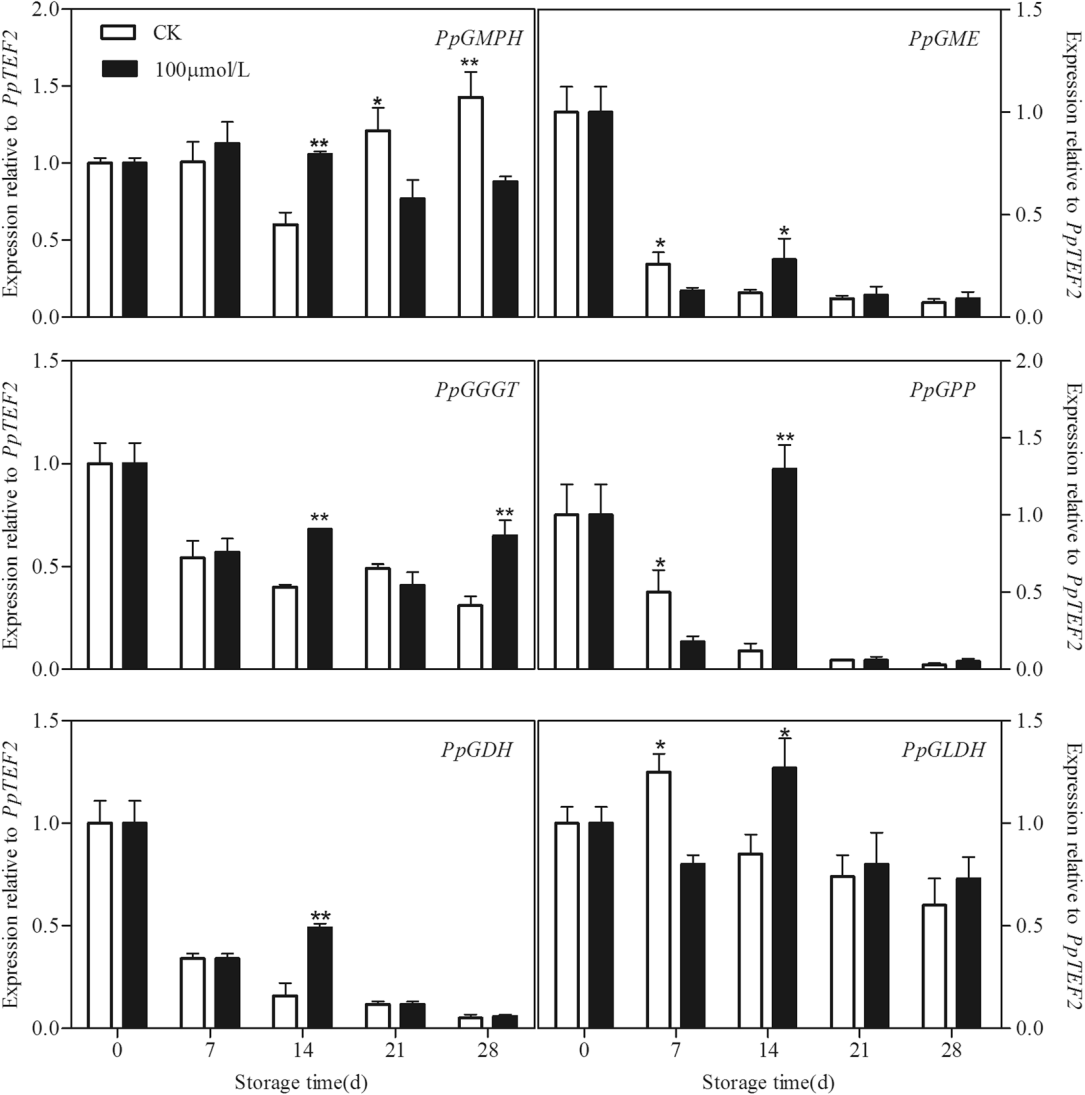
Effect of exogenous melatonin treatment on expression of AsA biosynthetic genes in peach fruit during storage at 4 °C. All data is presented as a mean of three biological replicates and error bars represent ± standard errors. Transcript abundance was determined using qPCR and was normalized using *PpTEF2*. GMPH, Mannose-1-phosphate guanylyltransferase. GME, GDP-D-mannose-3′,5′-epimerase. GGGT, GDP-L-galactose guanylyltransferase. GPP L-galactose-1-phosphate phosphatase. GDH, L-galactose-1-dehydrogenase. GLDH, L-galactono-1,4-lactone dehydrogenase. Asterisks (*) indicate significant differences between melatonin-treated and control samples (Student’s unpaired T test; *P < 0.05, **P < 0.01, and ***P < 0.001).

Degree of gene expression by *GMPH*, *GME*, *GGGT*, *GPP*, *GDH* and *GLDH* involved in AsA biosynthesis was analysed in peach fruit during cold storage. Expression of most of the AsA biosynthetic genes such as *PpGGGT*, *PpGME*, *PpGPP* and *PpGDH* decreased during the while storage in control fruit subjected to chilling stress. Transcripts of *PpGMPH* in control peaches declined firstly but increased after 21 days of storage but an increase in *PpGLDH* expression was observed at 7th of storage followed by a decline during the remaining time. Melatonin treatment upregulated the expression of all the biosynthetic genes at 14th day of storage. However, as compared to treated fruit, the control peaches showed a higher transcript of *PpGME*, *PpGPP* and *PpGLDH* at 7th and *PpGMPH* at 21st and 28th days of storage (Fig. 6).

## Discussion

It is well known that the major negative effect of chilling is that it causes oxidative stress in plants. At least some of the effects of chilling stress and tolerance mechanisms are believed to be mediated by chilling-induced H_2_O_2_^3,4^. H_2_O_2_ has been reported to operate as a signal, secondary messenger, and regulator of some gene expression for the activation of stress responses and defense pathways to regulate the cellular redox status^32,33^. Similarly to our previous study^29^, melatonin treatment at 100 µM alleviated chilling injury and induced tolerance against chilling in postharvest peach fruit during cold storage (Fig. 1a). A transient increase in H_2_O_2_ was suggested to signal activation of the protective mechanism for acclimation to chilling^34,35^. In our present study, melatonin-treated peach fruit also maintained a higher level of H_2_O_2_ than control ones at the initial time of cold stress (Fig. 1d), suggesting the transiently-induced H_2_O_2_ by melatonin may act as a diffusible signal molecule to up-regulate downstream defense genes encoding many antioxidants. Thus, H_2_O_2_ implicated an important contribution to the redox state of peaches, which ultimately played a crucial role in chilling tolerance^36^.

However, H_2_O_2_ plays a dual role in response to chilling and this response is dependent on the severity of the stress. When the generation of H_2_O_2_ exceeds the capacity of the plant to maintain cellular redox homeostasis, or when the production of H_2_O_2_ exceeds the capacity of the plant to scavenge them, the oxidative stress from lipid peroxidation, and oxidation of protein and DNA occurs^37–40^. Lipid peroxidation from excessive H_2_O_2_ leads to structural abnormalities and cell dysfunction^5^. Melatonin is a well-documented antioxidant and plays important roles in alleviating environmental stress-induced oxidation^20,22^. Exogenous application of melatonin has been found to improve plant chilling tolerance. Melatonin played a positive role in Bermudagrass to protect against cold stress through modulating photosynthesis and metabolism related pathways^41^. Melatonin could protect cucumber seeds and young seedlings against oxidative stress directly and indirectly detoxifying ROS, thereby plants grew better even in harmful environmental conditions^42,43^. Melatonin-treated rice seedlings inhibited the accumulation of ROS and alleviated the cold stress-induced inhibition to plant growth^44^. In present study, followed a transient induction of H_2_O_2_ content by melatonin, contents of H_2_O_2_ and MDA in peach fruit were significantly reduced by the melatonin treatment (Fig. 1b and c), indicating lower lipid peroxide and oxidative damage in the treated peaches. Our results suggested that melatonin treatment could reduce the chilling-induced oxidative stress and thus enhance tolerance against chilling in peach fruit. Similarly, application of melatonin improved plant resistance to cold stress in wheat seedlings in which H_2_O_2_ level was much lower than in non- treated ones^45^.

Plants have evolved enzymatic antioxidant system or the non-enzymatic antioxidant system to take control over the physiological ROS production. Among enzymatic antioxidant defense systems, SOD, the first line of defense against the oxidative stresses, dismutates O_2_^−^ to O_2_ and H_2_O_2_, while CAT can consequently convert H_2_O_2_ to H_2_O in plant cells^7^. Previous studies have established that melatonin promotes the activities of antioxidant enzymes to counteract the harmful effects caused by various environmental stresses. For instance, exogenous melatonin alleviated ROS accumulation and cold-induced oxidative damages by directly scavenging ROS and enhancing antioxidative enzymes in bermudagrass^18^. The applications of exogenous melatonin induced cold tolerance in rice seedlings by enhancing the activities of antioxidative enzymes^44^. Consistently, in our study, the high transcripts of *PpSOD*s and *PpCAT*s in melatonin-treated peach fruit further highlighted the role of melatonin in improving antioxidant capacity of postharvest peaches under chilling stress (Fig. 2).

Another important and efficient ROS-scavenging pathway is the AsA-GSH cycle, composed of antioxidant enzymes, including APX, monodehydroascorbate reductase (MDHAR), dehydroascorbate reductase (DHAR), and GR, and reducing substances, including AsA and reduced GSH^46^. After O_2_^−^ has been reduced by SOD to H_2_O_2_, APX catalyzes the conversion of H_2_O_2_ to monodihydroascorbate (MDHA) using AsA as substrate and thus removes toxic H_2_O_2_^47^. AsA and GSH can be recovered by a few relevant metabolic reactions catalyzed by MDHAR, DHAR and GR. Maintaining the efficient recycling of AsA and GSH via APX, MDHAR, DHAR and GR is crucial to maintaining the redox state at a high level so that they scavenge H_2_O_2_ efficiently^48,49^. There are several lines of evidence suggesting that promoting the AsA-GSH cycle contributes to the increase in cold tolerance of postharvest peaches^50,51^. Alleviation of chilling by hypobaric treatment was related to its effect on triggering the AsA-GSH cycle by up-regulating transcriptional expression of antioxidant related enzymes^52^. Melatonin has been also reported to increase the chilling tolerance in cucumber seedlings by accelerating the AsA-GSH cycle to enhance ROS scavenging ability^43^. In present study, accompanying with more AsA accumulation in melatonin-treated peaches (Fig. 3a), the transcript levels of genes in ASA-GSH cycle including *PpAPX*s, *PpMDHAR*s, *PpDHAR*s and *PpGR*s were also higher than in control fruit (Figs 4 and 5). Our results demonstrated that melatonin activated the expression of genes involved in ASA-GSH cycle in postharvest peaches under chilling stress, leading to the higher levels of AsA and subsequently lower H_2_O_2_ contents after the transient increase of H_2_O_2_ in the treated fruit (Figs 1a and 3a). This study confirmed that the AsA-GSH cycle plays an important role in implementing the function of exogenous melatonin in harvested peach fruit under chilling conditions.

In fact, it has been firmly established that melatonin is in favor of AsA production in plants under various stress conditions^18,41,53^. In agreement with these findings, despite the upregulation of all the genes involved in AsA-GSH cycle in peach fruit treated with melatonin, only higher AsA content was observed (Fig. 3a), which suggested that AsA may play a more dominant role in ROS scavenging than GSH in melatonin treated fruit. Therefore, the expression profiles of six L-galactose (Gal) pathway-related genes involved in AsA biosynthesis were also analysed in order to explain the higher AsA content in the treated peaches^54^. In our present study, expression of most of genes in control peach declined with storage time, indicating the loss of capacity to AsA accumulation under chilling stress. However, the transcripts of all these genes were up-regulated in peach fruit treated with melatonin (Fig. 6). It could thus be seen that melatonin might affect the AsA biosynthetic pathway in peaches subjected to chilling and consequently its final content during storage. To the best of our knowledge, this is the first report on the roles of the last six genes in the Gal pathway in AsA biosynthesis for chilling tolerance regulation in melatonin-treated peaches at the molecular level.

**Figure 7:**
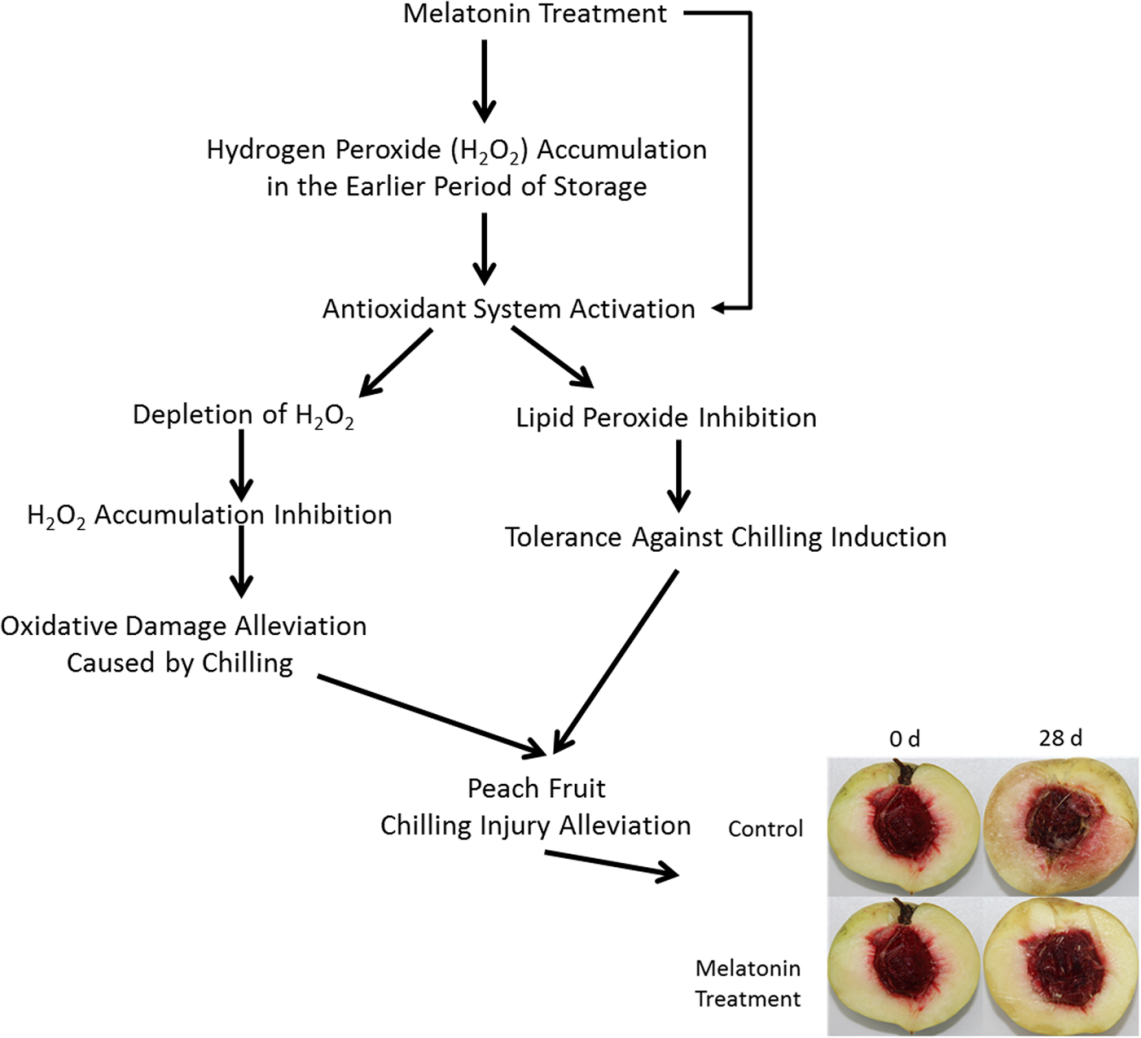
Proposed model for melatonin-mediated chilling tolerance in postharvest peach fruit.

Based on evidence provided herein, a novel model for melatonin and chilling tolerance in postharvest peaches subjected to chilling is proposed (Fig. 7). As mentioned above, treatment with melatonin induced chilling tolerance in peach after harvest. The higher level of H_2_O_2_ in melatonin treated peaches in the earlier period of storage might serve as a signal to activate antioxidant system and subsequently induce tolerance against chilling. When the chilling progressed, the activated antioxidant system in turn scavenged excess H_2_O_2_, thereby inhibited H_2_O_2_ accumulation and alleviated the oxidative damage caused by chilling in the treated peaches, which might be partially accountable for the induced chilling tolerance. However, it is well documented that melatonin can not only directly scavenge some ROS, but can also modulate antioxidant enzymes and enhance cellular antioxidants. Therefore, the induced transcripts of genes involved antioxidant systems and AsA biosynthesis was likely to the results of transiently increased-H_2_O_2_ induced by melatonin or the direct effect of melatonin or both. However, as a direct scavenger of ROS, why melatonin enhanced H_2_O_2_ accumulation at the initial storage time and which signal pathway was involved still remain unknown, which is deserved to further investigation.

## Methods

### Melatonin treatment

Peach fruit (*Prunus persica* Batsch cv. Hujing) were harvested at commercial maturity from a local orchard in Fenghua, China. Trees were subjected to standard horticultural practices and fruit ripening stage was defined according to the previous study^55^. Twenty fruit per each of the ten plants at 135 DAFB (days after full bloom) were picked and quickly transferred to the laboratory. The peaches were selected for uniformity without any damage and randomly divided into two groups. The first group was immersed into solutions of 100 µM melatonin for 120 min, whereas the second group of fruit was soaked in sterile deionized water for 120 min and considered as the control. Thereafter, all fruit were air-dried at room temperature for approximately 30 min, then transferred to 4 °C and 80% relative humidity for 28 d. Fruit samples were taken before melatonin treatment (time 0) and at 7 d intervals during storage for measurements of chilling injury index, levels of O_2_^−^, H_2_O_2_, AsA and GSH and molecular analysis. Each treatment was repeated three times, and the experiment was conducted twice.

### Chilling injury index

The degree of chilling was visually assessed on the mesocarp surface, following a double cut parallel to the axial diameter. The extent of flesh browning was divided into four classes: 0, no browning; 1, browning covering <25% of the cut surface; 2, browning covering ≥25% but <50% of cut surface; 3, browning covering ≥50%. Chilling injury index was calculated using the following formula:

Chilling injury index = ∑ [(browning level) × (number of fruit at the browning level)]/(total number of fruit in the treatment).

### MDA, O_2_^−^ and H_2_O_2_ analysis

To analysis MDA content, 2 g of flesh tissue was homogenized with 5 mL of 0.5% (w/v) trichloroacetic acid (TCA) and then centrifuged at 10,000 × g for 10 min at 4 °C. MDA content was determined and expressed as μg g^−1^ FW fresh weight (FW)^56^. O_2_^−^ production was measured following previously described procedures^57^. Briefly, 5 g of flesh tissue was ground in 5 ml of 50 mM phosphate buffer (pH 7.8). The homogenate was centrifuged at 10 000 × g for 20 min at 4 °C. The supernatant was used for the determination of O_2_^−^ production. O_2_^−^ production was calculated against the standard curve using sodium nitrite as a standard and expressed as nmol g^−1^ FW min^−1^. For H_2_O_2_ determination, 2 g of fresh tissue was homogenized with 5 mL of chilled 100% acetone and then centrifuged at 10 000 × g for 20 min at 4 °C. The supernatant was collected immediately for H_2_O_2_ analysis by a method based on titanium oxidation^58^. H_2_O_2_ concentration was determined by a standard graph constructed by direct addition of H_2_O_2_ to the titanium solution and expressed as mg g^−1^ FW.

### AsA and GSH analysis

To determine the AsA content, 2 g of flesh tissue was homogenized in ice-cold 5% (w/v) trichloroacetic acid (TCA) and then centrifuged at 10,000 × *g* for 20 min at 4°C. AsA content was measured following previously described procedures^59^. The results were expressed in μg g^−1^ FW. For GHS determination, each flesh tissue (2 g) was homogenized in 50 mM sodium phosphate buffer (pH 7.0) and then centrifuged at 10,000 × *g* for 20 min at 4 °C. The supernatant was used. GSH was assayed following previously described procedures^60^. The results were expressed in μg^−1^g FW.

### Total RNA extraction and cDNA synthesis

Frozen tissues were carefully grounded in liquid nitrogen. Total RNA was extracted using Plant RNA Kit (Omega Bio-Tek Inc., Norcross, GA, USA) according to the manufacturer’s instructions. The RNA was treated with amplification grade RNase-free DNase1 (Omega Bio-Tek Inc., Norcross, GA, USA) to remove any DNA contamination prior to cDNA synthesis. Reverse transcription (RT) was carried out using 2 µg of total RNA and the SuperRT First Strand cDNA Synthesis Kit (CWBIO, Beijing, China) as recommended by the manufacturer.

### Quantitative Real-Time PCR (qPCR)

For each primer pair, a fragment was amplified from cDNA and purified. A 10-fold dilution series of the fragment wa. s prepared over 7 orders of magnitude and used as a template for qRT-PCR in duplicate and generate standard curves. The reaction efficiency (E) of each primer pair was determined using the formula E = 10^−1^/slopeThe primers used for qRT-PCR are detailed in Table S1. QPCR reactions were performed with an Mx3000P qPCR System (Agilent Stratagene, Santa Clara, CA, USA) and the reactions consisted of 250 nM forward and reverse primer, SYBR Green PCR master mix (Thermo Fisher Scientific Inc., Pittsburgh, PA, USA), and 2 µl of cDNA template (diluted 1 in 5). Two-step qPCR analysis was performed, and the thermal cycling conditions consisted of an initial denaturation at 95 °C for 7 min, and then for 40 cycles as follows: denaturation at 95 °C for 15 s combined with each primer specific annealing temperature ranged from 50 °C to 60 °C for 30 s, then completed with a melting curve analysis program. QPCR data was calibrated relative to *PpTEF2* (JQ732180) expression level at zero time for each treatment, following the 2^−ΔΔCt^ method for relative quantification. Three independent biological replicates were analysed for each sample.

### Statistical analysis

Experiments were performed using a completely randomized design. All statistical analyses were performed with SPSS package program version 16.0 (SPSS Inc., Chicago, IL, USA). Data were analysed by one-way analysis of variance (ANOVA). Student’s unpaired *T* test was used to compare the means at P < 0.05.

## Electronic supplementary material

Primers Sequences Used for q-PCR and Amplicon Characteristics
