# Supplementary material for: Melatonin increases chilling tolerance in postharvest peach fruit by alleviating oxidative damage
Source: Sci Rep. 2018 Jan 16;8:806. doi: 10.1038/s41598-018-19363-5 (PMC5770464; doi:10.1038/s41598-018-19363-5)
Supplement: Supplementary file 1 — Primers Sequences Used for q-PCR and Amplicon Characteristics [file 41598_2018_19363_MOESM1_ESM.docx]

Melatonin increases chilling tolerance in postharvest peach fruit by alleviating oxidative damage

Shifeng Cao, Jiarong Shao, Liyu Shi, Liwei Xu, Ziming Shen, Wei Chen, Zhenfeng Yang,*

*Corresponding author Email: yangzf@zwu.edu.cn

College of Biological and Environmental Sciences, Zhejiang Wanli University, Ningbo, 315100, People’s Republic of China

**Table S1 Primers Sequences Used for q-PCR and Amplicon Characteristics**

| Gene name | GDR/NCBI accession No. | Forward and Reverse Primer Sequence [5'-3'] | Annealing Temperature/°C | Amplicon Size/bp |
| --- | --- | --- | --- | --- |
| *PpTEF2* | JQ732180 | F：GGTGTGACGATGAAGAGTGATG  R：TGAAGGAGAGGGAAGGTGAAAG | 59 | 129 |
| *PpSOD1* | ppa011882m | F：CATCGTAACTATGTGGAAGGT  R：TGAGCAGCGTTGTTGAAT | 56 | 134 |
| *PpSOD2* | ppa008765m | F：GGAGACCTTAGAATATCACT  R：TGTTGTATGTAGCAAGTATG | 52.3 | 125 |
| *PpSOD3* | ppa010748m | F：AGAATCTTACTCCTGTTG  R：CACTAATGCTTCCATAGA | 59 | 98 |
| *PpSOD4* | ppa008639m | F：CTACGACAACTGCCATAG  R：AAGAGGAAGACGAAGAGA | 54.7 | 75 |
| *PpSOD7* | ppa012845m | F：GGACCACAATCAATCATT  R：AGACCAATAATACCACAAG | 50 | 131 |
| *PpSOD8* | ppa013403m | F：TTCCATATTCACGCTCTT  R：GAATCTGCCAGTCCTTAA | 49.9 | 85 |
| *PpCAT1* | ppa004776m | F：GCAGAGAATGAACAACTT  R：GAGTATCAGAATAGGAGAAGA | 55.5 | 106 |
| *PpCAT2* | ppa004763m | F：AATCTTCGCATATTCTGA  R：TTATGATGAGGACACTTG | 51.5 | 90 |
| *PpAPX1* | ppa010673m | F：CCATCAAGCAACAGTTCC  R：CAGTAATCTCAACAGCAACA | 51.4 | 78 |
| *PpAPX2* | ppa010426m | F：TATCAGAAGGCAGTGGAT  R：GCTAATCGGAGAATTATAGGA | 50 | 86 |
| *PpAPX3* | ppa009582m | F：ATCGTCTAGTGTCAAGGA  R：TTCGTATAAGTAACTCAGGAT | 52 | 106 |
| *PpAPX4* | ppa009538m | F：TGTATGCGAAGGATGAAGAT  R：TGTTAGTAGAAGAGGAGATTGG | 53.4 | 99 |
| *PpAPX6* | ppa006270m | F：GTAACATACGCAGACTTG  R：CCATACTTCATAGGAATCTT | 54.6 | 83 |
| *PpAPX7* | ppa015878m | F：CACTGCCATTGCTTCTTC  R：TATTCACTTATATCCGACTCTACC | 57 | 79 |
| *PpAPX8* | ppa008008m | F：AGTCTCCTTCCTATCAAC  R：TATCCTCATTAGCAGTATCA | 59 | 146 |
| *PpMDHAR1* | ppa005968m | F：TCAGATGGAGAGGTCAAG  R：AACACCAACAACAACAATG | 52.3 | 75 |
| *PpMDHAR2* | ppa005081m | F：AGAGACCATAAGTTACAA  R：GAAGATTCCATCAAGTTG | 48 | 153 |
| *PpDHAR2* | ppa011390m | F：TGATGTTCTTGGCGACTG  R：CTCTGGATTCACTTCGGTAA | 59.4 | 130 |
| *PpDHAR3* | ppa010038m | F：GTTGGTGGACTTGGCTAA  R：CAAAGGTGGATCTGGATACT | 49.7 | 148 |
| *PpGR1* | ppa004670m | F：AGAACAGGCAATAGAACA  R：CTCAGCATCAACAAGAAG | 49.8 | 124 |
| *PpGR2* | ppa003678m | F：ATGGTTCGTTGTCTCTGA  R：CCTACTTCCTCCAATCCTAA | 55.5 | 112 |
| *PpGMPH* | ppa007618m | F：TGCTGGAATATACCTGTTGA  R：AAGGACCATTGCGAAGAG | 52 | 121 |
| *PpGME* | ppa007264m | F：TGAGATGGCGGAGATAGT  R：TGTGTTGTCTGAGTTACGA | 58.2 | 100 |
| *PpGGGT* | ppa005736m | F：GAAGCAAGCGAAGATGGT  R：CGAGGAATCAACAGCACAT | 54.4 | 125 |
| *PpGPP* | ppa009952m | F：GTGGTGCTGTGATTGTTAC  R：AAAGGGTTTGATGCTGCTA | 60 | 100 |
| *PpGDH* | ppa008631m | F：AAGGTGTTGGTGTCATTAGT  R：CACGCAGACTTCAATTCAG | 54.4 | 103 |
| *PpGLDH* | ppa025367m | F：CGATGGATGAACAGGTGAT  R：CTCTCAACGCACTGGATG | 63 | 148 |
